# Influence of genetic polymorphisms on oral health-related quality of life after root canal treatment

**DOI:** 10.1590/0103-6440202405678

**Published:** 2024-03-22

**Authors:** Graciane E. R. Queiroz, Thuanny Castilho, Ludmila Silva Guimarães, Vania Gomes Moraes, Erlange Andrade Borges da Silva, Erika Calvano Küchler, Alice Corrêa Silva-Sousa, Manoel Damião Sousa-Neto, Lívia Azeredo Alves Antunes, Leonardo Santos Antunes

**Affiliations:** 1 Postgraduate Program, School of Dentistry, Fluminense Federal University, Niterói, RJ, Brazil.; 2 Postgraduate Program, School of Dentistry, Fluminense Federal University, Nova Friburgo, RJ, Brazil.; 3 Tuiuti of Paraná University, Curitiba, Brazil.; 4 Restorative Dentistry Department, School of Dentistry of Ribeirão Preto, University of São Paulo, Ribeirão Preto, SP, Brazil.; 5 Specific Formation Department, School of Dentistry of Nova Friburgo, Fluminense Federal University, Nova Friburgo, RJ, Brazil.

**Keywords:** Genetic polymorphism, oral health-related quality of life, OHRQoL, root canal preparation.

## Abstract

To evaluate the impact of genetic polymorphisms in interleukins (IL1A rs17561, rs1304037; IL10 rs1800871; IL1RN rs9005), nitric oxide (NOS2 rs2779249, rs2897518) and suppressor of cytokine signaling (SOCS1 rs243327, rs33977706) on oral health-related quality of life (OHRQoL) of patients under-going root canal treatment (RCT). Methods: The sample consisted of 108 participants, presenting single-rooted teeth with asymptomatic periapical periodontitis. The impact of the OHRQoL was recorded using the Oral Health Impact Profile (OHIP-14) before, seven, and 30 days after RCT. Saliva samples were collected as a source of genomic DNA. Genetic polymorphisms were genotyped by Real-Time PCR using the Taqman method. Univariate and Multivariate analyses were used (p<0.05). Results: A significant difference was observed for the polymorphism rs2297518 in the NOS2 gene in functional limitation in the codominant (p=0.037) and recessive (p=0.001) models; in the physical pain (p<0.001 in both models); in psychological discomfort (p<0.001 in both models); in physical disability (p<0.001 in both models) and in psychological disability (p<0.001 in both models). Polymorphisms in the SOCS1 gene, in the recessive model, rs33977706 (p=0.045) and rs243327 (p=0.019), influenced the OHRQoL in the psychological discomfort domain. Conclusions: Polymorphisms in NOS2 and SOCS1 genes influenced the OHRQoL of patients undergoing RCT.

## Resumo

Avaliar o impacto de polimorfismos genéticos em interleucinas (IL1A rs17561, rs1304037; IL10 rs1800871; IL1RN rs9005), óxido nítrico (NOS2 rs2779249, rs2897518) e supressor da sinalização de citocinas (SOCS1 rs243327, rs33977706) na qualidade de vida relacionada à saúde bucal (QVRSB) de pacientes submetidos a tratamento endodôntico (TE). Métodos: A amostra foi composta por 108 participantes, que apresentavam dentes unirradiculares com lesão periapical assintomática. O impacto da QVRSB foi registrado usando o Oral Health Impact Profile (OHIP-14) antes, sete e 30 dias após o TE. Amostras de saliva foram coletadas como fonte de DNA genômico. Os polimorfismos genéticos foram genotipados por PCR em tempo real usando o método Taqman. Análises univariadas e multivariadas foram utilizadas (p<0,05). Resultados: Observou-se diferença significativa para o polimorfismo rs2297518 no gene NOS2 na limitação funcional nos modelos codominante (p=0,037) e recessivo (p=0,001); na dor física (p<0,001 em ambos os modelos); no desconforto psicológico (p<0,001 em ambos os modelos); na deficiência física (p<0,001 em ambos os modelos) e na deficiência psicológica (p<0,001 em ambos os modelos). Polimorfismos no gene SOCS1, no modelo recessivo, rs33977706 (p=0,045) e rs243327 (p=0,019), influenciaram a QVRSB no domínio desconforto psicológico. Conclusões: Polimorfismos nos genes NOS2 e SOCS1 influenciaram a QVRSB de pacientes submetidos a TE.

## Introduction

Apical periodontitis arises from an inflammatory response as a defense mechanism of the body against microorganisms present in the infected root canal, leading to chronic inflammation ^1^. Individual genetic factors may also be involved in this etiology ^2^. Proper instrumentation, comprehensive disinfection, and obturation are critical to successful root canal treatment. In this context, healing of the periapical tissues is also necessary to contribute to proposed treatment success ^3^. However, pain after root canal treatment can occur, being one of the most common side effects, affecting from 3% to 58% of the population ^4^. 

Traditional clinical indicators are not instruments that provide complete information to determine the oral health-related quality of life (OHRQoL); therefore, socio-dental indicators are used as a complement to obtain the psychological and social range affected by oral health ^5^. There are some instruments in the literature that measure OHRQoL. Oral Health Impact Profile (OHIP) is an instrument commonly used to assess the quality of life of oral health in adults. The OHIP-14 collection instrument is an indicator to present a measure of disability, discomfort, and disadvantage attributed to the oral condition ^6^. Previous studies demonstrated that endodontic factors affect OHRQoL ^7^. Specifically, post-endodontic pain can cause changes in the patient's oral health-related quality of life (OHRQoL) ^8^.

The Consortium for Genetics and Quality of Life Research (GeneQoL) developed a list of potential biological markers, such as candidate genes, involved in quality-of-life outcomes ^9^ ^,^ ^10^. Among the candidate genes reported by the GeneQoL consortium study, interleukin 1 alpha (*IL1A*), interleukin 10 (*IL10*), and interleukin 1 receptor antagonist (*IL1RN*) were associated with health-related quality of life (HRQoL), symptoms related to general health, such as pain, fatigue, and emotional and social functioning ^10^. Nitric oxide pathway genes are involved in neuropsychiatric disorders ^11^ ^,^ ^12^ and pain and inflammatory diseases ^13^ ^,^ ^14^ ^,^ ^15^. Inducible nitric oxide (NOS2), through the metabolic pathway, can also be associated with HRQoL in the domain of emotional functioning (depression) ^10^. In dental research, some studies have already reported the association between genetic polymorphisms and OHRQoL in patients with temporomandibular disorders ^16^, dental caries ^17^ ^,^ ^18^, open bite malocclusion ^19^, and patients submitted to orthognathic surgery ^20^.

To the best of our knowledge, there are no studies in the dental field assessing the impact of genetic polymorphisms on the OHRQoL of patients undergoing root canal treatment. Furthermore, it is necessary to explore genetic biomarkers for OHRQoL also in patients submitted to dental treatment. Therefore, in this study, we evaluated whether genetic polymorphisms in *IL1A, IL10, IL1RN, NOS2,* and *SOCS1* genes are potential biomarkers for OHRQoL in patients undergoing root canal treatment in teeth with asymptomatic periapical periodontitis. The hypothesis tested was that genetic polymorphism in *IL1A, IL10, IL1RN, NOS2, and SOCS1* genes can modulate OHRQoL in endodontically treated patients. 

## Material and Methods

### Ethical approval, type of study, and sampling

This cohort study was written following the guidelines of the Strengthening the Study Statement Reporting of the Genetic Association Checklist ^21^. The research was approved by the Local Human Ethics Committee (5.545.749). All participants consented to participate in the research through the informed consent form.

The study population was composed of 108 patients treated at the Dental Clinic of Health Institute of Nova Friburgo/Fluminense Federal University, from July 2022 to July 2023. Patients over 18 years old, who had at least one single-rooted tooth for root canal treatment, with periapical periodontitis, without preoperative pain or edema were included. On the other hand, patients with systemic disorders, allergic to sodium hypochlorite, allergic to the drug Ibuprofen, who used antibiotics in the last 30 days, when it was not possible to perform foraminal patency, patients requiring endodontic retreatment, who presented vital teeth and teeth with very wide apical foramen were excluded from the research. 

### Treatment protocol

The proposed root canal treatment was performed by a specialist in endodontics (L.S.G) with ten years of experience, following the same protocol for all study participants ^22^. Initially, clinical evaluation, periapical radiography, and sensitivity tests were performed. Then local anesthesia with 2% lidocaine with 1:100,000 epinephrine (Alphacaine; DFL Indústria e Comércio Ltda, Taquara, Rio de Janeiro, Brazil) was administered. Access to the pulp chamber was prepared with a sterile diamond bur (KG Sorensen, Cotia, São Paulo, Brazil) at high speed. The tooth was then isolated with a compatible clamp and rubber dam and disinfected with 2.5% NaOCl (Fórmula & Ação, São Paulo, Brazil). Initial irrigation was performed with 2.5% sodium hypochlorite (Formula & Ação, São Paulo, Brazil) in the pulp chamber, and the root canal was continuously flooded with the solution. Each tooth was irrigated with the same volume of irrigant, 15 ml of 2.5% NaOCl (Fórmula & Ação), using a 30-G irrigation needle (Max-i-Probe; Dentsply Sirona, York, Pennsylvania, USA). 

To establish the WL, an electronic apex locator, RomiApex A-15 (Romidan, Kiryat Ono, Israel), was used. The working length was measured with a 15 K-file (Dentsply Sirona, York, Pennsylvania, USA) and patency was maintained with K-file 10 (Dentsply Sirona) during the entire instrumentation. Mechanized instrumentation was then performed with a reciprocating system, following the manufacturer's instructions.

Final irrigation with 17% EDTA for 5 minutes, neutralization with 2 ml of saline solution, and drying of the root canals with a sterile cone were performed. No mechanical agitation was performed in the irrigating solution and EDTA.

Finally, filling using the lateral condensation technique with a gutta-percha cone R40 or R50 and MTA Fillapex (Angelus, Londrina, Paraná, Brazil) was performed. A definitive restoration was done, and the occlusion was checked and adjusted.

### Non-clinical data assessment

To assess the impact of OHRQoL, the OHIP-14 questionnaire was used, which consists of a structured instrument valid in Portuguese ^23^. The OHIP-14 consists of two questions for each of the seven domains: functional limitation, physical pain, psychological discomfort, physical disability, psychological disability, social disability, and handicap. The set of OHIP-14 responses was evaluated based on a five-point Likert scale (0 = never; 1 = hardly ever; 2 = occasionally; 3 = fairly often; and 4 = very often), with study participants being allowed to select one of the five options ^6^. The total scale score was calculated by adding the points for each OHIP-14 item, ranging from 0 to 56 points. High scores indicated a negative impact on OHRQoL.

The OHIP-14 questionnaire was administered by a pre-trained professional who did not take part in the clinical procedure (E.A.B.S) and was answered by each patient in three moments: before the procedure, seven days after the root canal treatment, and 30 days after the procedure.

### Predictor variables

The independent variables gender, ethnicity, tooth length, arch position (mandible or maxilla), medication, edema, and periapical lesion area were adjusted and used in the multivariate analysis.

The initial radiograph of each case was entered into the ImageJ/Fiji 1.46 software (http://imagej.nih.gov/ij/) and the area of the periapical lesion was determined by a single operator (E.A.B.S) with specific software tools, considering the area of the lesion in mm^2^.

To determine the length of the tooth during treatment, the apex locator was used. Thereafter, all teeth included in the research were standardized from the cemento-enamel junction to the apex of the tooth with the tools available in Kodak Digital software (Kodak, Rochester, New York, USA) (E.A.B.S).

### Biological material collection and molecular analysis

Genomic DNA for genotyping analysis was extracted from buccal cells isolated from saliva. The patients performed a mouthwash with 15 ml of 5% saline solution for 1 minute, the content was stored in 50 ml centrifuge tubes. The samples were kept at -20◦C for subsequent DNA extraction, using a method previously described in the literature ^24^.

The polymorphisms in *IL1A* (rs17561 and rs1304037), *IL10* (rs1800871), *IL1RN* (rs9005), *NOS2* (rs2779249 and rs2297518), and *SOCS1* (rs243327 and rs33977706) genes were chosen because they are potential candidates for the study of OHRQoL ^16^ ^,^ ^17^ ^,^ ^25^. These polymorphic genetic variations were selected and evaluated using USCS Genome Bioinformatics. Polymorphisms were genotyped by real-time PCR using the TaqMan method. Data interpretation was performed using software provided by Applied Biosystems (Foster City, CA, USA) for allelic discrimination. Laboratory examiners were blinded to the sample group.

### Statistical analysis

The median and interquartile interval of the scores were reported for each day and each genotype. Univariate and Multivariate Linear regression by Generalized Estimating Equations (GEE) was applied to analyze the differences between genotypes. The SNPs that were close to the p<0.05 in Univariate analysis were analyzed in Multivariate Linear Regression adjusted by the associated factors with each dominium. All analyses were performed by IBM SPSS version 25.0 (IBM Corp. Armonk, USA), and values of p<0.05 indicate statistical difference.

The Hardy-Weinberg equilibrium was assessed by the Pearson chi-square test.

## Results

**Figure 1:**
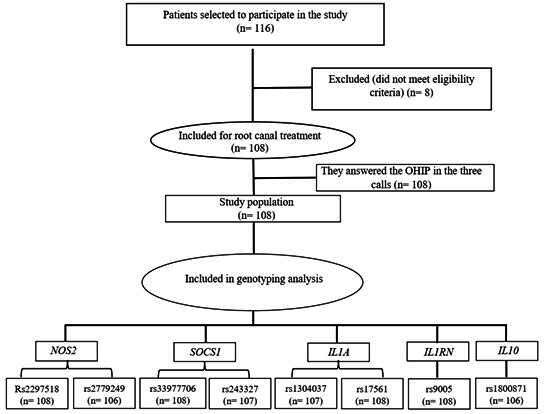
Flowchart of the genotyping analysis.

A total of 108 patients of both sexes, 38 men and 70 women, with a mean age of 40.5 years (Standard Deviation (SD) 13.2), requiring root canal treatment in necrotic teeth and asymptomatic periapical periodontitis, were included in this study. Only single-rooted teeth were treated, totaling 69 dental elements in the maxilla and 39 in the mandible. A flowchart of the genotyping analysis is shown in Figure 1. 

The overall mean OHIP-14 score recorded was 17.00 (SD 11.70), 3.96 (SD 6.87), and 2.32 (SD 4.49), while the median scores were 14.00 (0.00-52.00), 1.00 (0.00-36.00) and 0.00 (0.00-30.00), before, 7 and 30 days after root canal treatment, respectively.

The genotypic distribution of each genotype was consistent with the Hardy-Weinberg equilibrium proportions (Table 1). There was no significant difference between the genetic polymorphisms in the genes analyzed and the total score of the OHIP-14 instrument for OHRQoL (Table 2). The associations between genotypes and each domain of the OHIP-14 instrument in the three different models (co-dominant, recessive, and dominant) supplementary data may be requested by correspondence to authors. The analysis of the rs2297518 polymorphism in *NOS2* gene revealed significant difference in the respective domains: functional limitation [co-dominant (p=0.037) and recessive (p= 0.001) models]; physical pain [co-dominant (p<0.001) and recessive (p<0.001) models]; psychological discomfort [co-dominant (p<0.001) and recessive (p<0.001) models]; physical disability [co-dominant (p<0.001) and recessive (p<0.001) models]; and psychological disability [co-dominant (p<0.001) and recessive (p<0.001) models]. 

**Table 1:** Details of the SNPs investigated.

Gene	Location	SNP ID	Reference SNP alleles	Alleles minimal frequency
NOS2	Chr.17: 27801555	rs2779249	C/A	A=0.27
	Chr.17: 27769571	rs2297518	A/G	A=0.17
SOCS1	Chr.16: 11259447	rs243327	A/G	G=0.39
	Chr.16: 11256298	rs33977706	A/C	A=0.17
IL1A	Chr.2: 112779646	rs17561	A/C	A=0.22
	Chr.2: 112774659	rs1304037	C/T	C=0.28
IL1RN	Chr.2:113133835	rs9005	A/G	A=0.32
IL10	Chr.1: 206773289	rs1800871	A/G	A=0.43

Notes: Obtained from the database: http://www.thermofisher.com; ID: identification; SNP: single-nucleotide polymorphisms; Chr: Chromosome.

**Table 2:** Impact of genetic polymorphisms on the total score of OHIP-14 before, 7, and 30 days after root canal treatment.

Total score														
Gene	SNP	Model	Genotype	Before treatment			7 days after			30 days after				
				Med.	25th	75th	Med.	25th	75th	Med.	25th	75th	β	P value
*NOS2*	rs2779249	Co-Dominant	CC	14.00	8.0	24.0	1.00	0.0	2.0	1.00	0.0	3.0	Ref.	Ref.
			AC	15.50	10.0	23.5	0.50	0.0	4.0	0.00	0.0	2.0	-0.78	0.500
			AA	15.00	6.5	27.5	4.00	0.0	6.0	0.00	0.0	4.0	0.95	0.685
		Dominant	CC	14.00	8.0	24.0	1.00	0.0	2.0	1.00	0.0	3.0	Ref.	Ref.
			AC + AA	15.50	9.0	24.0	1.00	0.0	4.0	0.00	0.0	2.0	-0.42	0.717
		Recessive	CC + AC	14.50	8.0	24.0	1.00	0.0	4.0	0.00	0.0	3.0	Ref.	Ref.
			AA	15.00	6.5	27.5	4.00	0.0	6.0	0.00	0.0	4.0	1.34	0.554
	rs2297518	Co-Dominant	GG	14.00	7.0	26.0	1.00	0.0	4.0	0.00	0.0	2.0	Ref.	Ref.
			AG	14.00	8.0	21.0	2.00	0.0	4.5	1.00	0.0	3.0	-0.37	0.744
			AA	16.00	16.0	16.0	6.00	6.0	6.0	2.00	2.0	2.0	0.17	0.807
		Dominant	GG	14.00	7.0	26.0	1.00	0.0	4.0	0.00	0.0	2.0	Ref.	Ref.
			AG + AA	14.00	8.0	21.0	2.00	0.0	5.0	1.00	0.0	3.0	-0.35	0.752
		Recessive	GG + AG	14.00	8.0	24.0	1.00	0.0	4.0	0.00	0.0	3.0	Ref.	Ref.
			AA	16.00	16.0	16.0	6.00	6.0	6.0	2.00	2.0	2.0	0.27	0.642
*SOCS1*	rs243327	Co-Dominant	AA	15.50	10.0	25.0	2.00	0.0	4.0	0.00	0.0	2.0	Ref.	Ref.
			AG	13.00	5.0	19.0	1.00	0.0	3.0	0.00	0.0	2.0	-1.65	0.141
			GG	17.00	7.0	33.0	2.00	0.0	6.0	2.00	0.0	6.0	1.80	0.273
		Dominant	AA	15.50	10.0	25.0	2.00	0.0	4.0	0.00	0.0	2.0	Ref.	Ref.
			AG + GG	14.00	7.0	21.0	1.00	0.0	4.0	0.00	0.0	3.0	-0.27	0.802
		Recessive	AA + AG	14.00	8.0	21.5	1.00	0.0	4.0	0.00	0.0	2.0	Ref.	Ref.
			GG	17.00	7.0	33.0	2.00	0.0	6.0	2.00	0.0	6.0	2.64	0.095
	rs33977706	Co-Dominant	CC	16.00	9.0	24.0	2.00	0.0	4.0	0.00	0.0	2.0	Ref.	Ref.
			AC	13.50	5.0	21.0	0.50	0.0	3.5	0.00	0.0	2.5	-0.04	0.974
			AA	14.00	8.0	26.0	2.00	0.0	5.0	2.00	0.0	4.0	1.40	0.328
		Dominant	CC	16.00	9.0	24.0	2.00	0.0	4.0	0.00	0.0	2.0	Ref.	Ref.
			AC + AA	14.00	7.0	22.0	1.00	0.0	4.0	1.00	0.0	3.0	0.45	0.679
		Recessive	CC + AC	14.00	8.0	24.0	1.00	0.0	4.0	0.00	0.0	2.0	Ref.	Ref.
			AA	14.00	8.0	26.0	2.00	0.0	5.0	2.00	0.0	4.0	1.43	0.320
*IL1A*	rs17561	Co-Dominant	CC	14.50	7.0	25.0	1.00	0.0	4.0	0.00	0.0	2.0	Ref.	Ref.
			AC	14.00	9.0	26.0	2.00	0.0	5.0	0.00	0.0	3.0	-0.07	0.950
			AA	14.00	4.0	17.0	0.00	0.0	6.0	2.00	0.0	2.0	-2.40	0.167
		Dominant	CC	14.50	7.0	25.0	1.00	0.0	4.0	0.00	0.0	2.0	Ref.	Ref.
			AC + AA	14.00	8.0	22.0	1.00	0.0	5.0	0.00	0.0	3.0	-0.37	0.749
		Recessive	CC + AC	14.00	8.0	25.0	1.00	0.0	4.0	0.00	0.0	3.0	Ref.	Ref.
			AA	14.00	4.0	17.0	0.00	0.0	6.0	2.00	0.0	2.0	-2.36	0.142
	rs1304037	Co-Dominant	TT	14.00	7.0	24.0	2.00	0.0	4.0	0.00	0.0	2.0	Ref.	Ref.
			TC	15.00	8.0	26.0	1.00	0.0	4.0	0.00	0.0	3.0	-0.27	0.832
			CC	12.00	8.0	17.0	1.00	0.0	6.0	2.00	0.0	5.0	-0.97	0.644
		Dominant	TT	14.00	7.0	24.0	2.00	0.0	4.0	0.00	0.0	2.0	Ref.	Ref.
			TC + CC	14.50	8.0	22.0	1.00	0.0	4.0	0.00	0.0	3.0	-0.39	0.753
		Recessive	TT + TC	14.50	8.0	24.5	1.00	0.0	4.0	0.00	0.0	2.0	Ref.	Ref.
			CC	12.00	8.0	17.0	1.00	0.0	6.0	2.00	0.0	5.0	-0.14	0.671
*IL1RN*	rs9005	Co-Dominant	GG	13.00	8.0	22.5	2.00	0.0	4.0	0.00	0.0	2.0	Ref.	Ref.
			AG	14.00	8.0	22.0	1.00	0.0	5.0	0.00	0.0	3.0	-0.12	0.913
			AA	20.00	6.0	26.0	0.00	0.0	11.0	0.00	0.0	2.0	1.42	0.532
		Dominant	GG	13.00	8.0	22.5	2.00	0.0	4.0	0.00	0.0	2.0	Ref.	Ref.
			AG + AA	16.00	8.0	25.0	1.00	0.0	5.5	0.00	0.0	3.0	0.18	0.870
		Recessive	GG + AG	14.00	8.0	22.0	1.00	0.0	4.0	0.00	0.0	3.0	Ref.	Ref.
			AA	20.00	6.0	26.0	0.00	0.0	11.0	0.00	0.0	2.0	1.48	0.502
*IL10*	rs1800871	Co-Dominant	GG	15.00	11.0	21.5	2.00	0.0	5.0	0.00	0.0	3.0	Ref.	Ref.
			AG	14.00	6.0	26.0	0.50	0.0	4.0	0.00	0.0	2.0	-1.73	0.128
			AA	11.00	7.0	26.0	1.50	0.0	13.0	2.00	0.0	5.0	0.56	0.842
		Dominant	GG	15.00	11.0	21.5	2.00	0.0	5.0	0.00	0.0	3.0	Ref.	Ref.
			AG + AA	14.00	6.0	26.0	1.00	0.0	4.0	0.00	0.0	2.0	-1.34	0.251
		Recessive	GG + AG	15.00	8.0	24.0	1.00	0.0	4.0	0.00	0.0	2.0	Ref.	Ref.
			AA	11.00	7.0	26.0	1.50	0.0	13.0	2.00	0.0	5.0	1.47	0.590

Notes: Univariate Linear Regression by Generalized Estimating Equations was performed to obtain the p-value. * means statistical significance (p<0.05). Med = Median.

In the multivariate linear regression by analysis of Generalized Estimating Equations, the rs2297518 polymorphism in *the NOS2* gene revealed a significant difference in the respective domains: functional limitation [co-dominant model CI = 0.23 (0.06-0.40), p<0.006 and recessive model CI = 0.28 (0.14-0.42), p<0.001]; physical pain [co-dominant model CI = -0.89 (-1.19 - -0.59), p<0.001 and recessive model CI = -0.81 (-1.08 - -0.54), p<0.001]; physical disability [co-dominant model CI = -0.90 (-1.20 - -0.60), p<0.001 and recessive model CI = -0.88 (-1.16 - -0.59), p<0.001]; and psychological disability [co-dominant model CI = 0.29 (0.22 - 0.37), p<0.001 and recessive model CI = 0.28 (0.21 - 0.34), p<0.001]. The rs243327 and rs33977706 polymorphisms in *the SOCS1* gene revealed significant differences in the psychological discomfort domain of OHIP-14 in the recessive model [CI = 0.63 (0.10 - 1.16), p = 0.019; CI = 0.55 (0.01 - 1.10), p = 0.045, respectively). Data referring to multivariate linear regression are presented in Table 3.

There was no significant difference between the polymorphisms in the *IL1A* (rs17561, rs1304037), *IL10* (rs1800871), *IL1RN* (rs9005), and *NOS2* (rs2779249) genes, and the total score and each domain of the OHIP-14 instrument in all analyzes carried out. 

**Table 3:** Multivariate Linear Regression by Generalized Estimating Equations analysis.

Domain	Genes	SNPs	Model	Reference	Genotype	Β (CI^†^ 95%)	P-Value	Adjusted by
Functional Limitation	*NOS2*	rs2297518	Co-Dominant	GG	AG	-0.21 (-0.46 - 0.03)	0.093	Tooth length
					AA	0.23 (0.06 - 0.40)	0.006*	
			Recessive	GG	AG + AA	0.28 (0.14 - 0.42)	<0.001*	
Physical pain	*NOS2*	rs2297518	Co-Dominant	GG	AG	-0.44 (-0.90 - 0.06)	0.053	Gender, medication, edema, and periapical lesion area
					AA	-0.89 (-1.19 - -0.59)	<0.001*	
			Recessive	GG	AG + AA	-0.81 (-1.08 - -0.54)	<0.001*	
	*IL10*	rs1800871	Co-Dominant	GG	AG	-0.38 (-0.79 - 0.03)	0.074	
					AA	-0.21 (-0.84 - 0.41)	0.503	
Psychological discomfort	*NOS2*	rs2297518	Co-Dominant	GG	AG	-0.07 (-0.68 - 0.53)	0.808	Gender, medication, edema, and arch position
					AA	0.42 (-0.01 - 0.85)	0.053	
			Recessive	GG	AG + AA	0.44 (0.06 - 0.82)	0.022*	
	*SOCS1*	rs243327	Recessive	AA	AG + GG	0.63 (0.10 - 1.16)	0.019*	
		rs33977706	Recessive	CC	AC + AA	0.55 (0.01 - 1.10)	0.045*	
Physical disability	*NOS2*	rs2297518	Co-dominant	GG	AG	-0.12 (-0.58 - 0.32)	0.579	Gender. medication, edema, and periapical lesion area
					AA	-0.90 (-1.20 - -0.60)	<0.001*	
			Recessive	GG	AA	-0.88 (-1.16 - -0.59)	<0.001*	
Psychological disability	*NOS2*	rs2297518	Co-dominant	GG	AG	0.05 (-0.08 - 0.18)	0.429	Periapical lesion area and arch position
					AA	0.29 (0.22 - 0.37)	<0.001*	
			Recessive	GG	AG + AA	0.28 (0.21 - 0.34)	<0.001*	
	*IL10*	rs1800871	Co-Dominant	GG	AG	-0.09 (-0.19 - 0.01)	0.073	
					AA	-0.06 (-0.30 - 0.18)	0.617	
Social disability	*SOCS1*	rs243327	Co-Dominant	AA	AG	-0.02 (-0.12 - 0.07)	0.629	Periapical lesion area and medication
					GG	0.01 (-0.05 - 0.19)	0.271	
			Recessive	AA	AG + GG	0.06 (-0.05 - 0.17)	0.302	
		rs33977706	Co-Dominant	CC	AC	0.05 (-0.05 - 0.16)	0.306	
					AA	0.08 (-0.04 - 0.21)	0.214	
			Dominant	CC	AC + AA	0.06 (-0.02 - 0.15)	0.157	
handicap	*SOCS1*	rs243327	Co-Dominant	AA	AG	-0.29 (-0.67 - 0.08)	0.124	Gender
					GG	0.14 (-0.39 - 0.67)	0.608	
Total Score	*SOCS1*	rs243327	Co-Dominant	AA	AG	-2.19 (-4.50 - 0.10)	0.062	Gender, ethnicity, medication, edema, and periapical lesion area
					GG	2.14 (-0.39 - 4.68)	0.098	

Notes: The analysis was performed with each genotype individually adjusted by the factors indicated in the last column. * Means P < 0.05. †CI means confidence interval.

## Discussion

The present study aimed to evaluate the impact of genetic polymorphisms of interleukins, suppressors of cytokine signaling, and nitric oxide on OHRQoL of patients undergoing root canal treatment in teeth with asymptomatic periapical periodontitis. The results rejected partially the presented hypothesis. Polymorphisms in the *NOS2* (rs2297518) and *SOCS1* (rs33977706 and rs243327) genes modulated the impact on OHRQoL, while polymorphisms in the *IL1A* (rs17561, rs1304037), *IL10* (rs1800871), *IL1RN* (rs9005) and *NOS2* (rs2779249) genes did not influence any domain on OHRQoL. To the best of our knowledge, this is the first study to address these genes in this outcome, which may contribute to a better clinical decision, since results based on the patient's self-perception of OHRQoL provide an important opportunity to complement clinical data ^26^ ^,^ ^27^. Molecular markers are involved in quality-of-life domains ^10^ and may be involved in OHRQoL domains.

Oral health includes the ability to speak, smile, smell, taste, touch, chew, swallow, and convey emotions through facial expressions with confidence and without pain, discomfort, or disease of the craniofacial complex ^28^. The OHIP14 is a quality-of-life assessment tool widely used in epidemiological studies. In the present study, the OHIP-14 instrument was used in its valid Portuguese version to assess the quality of life-related to oral health. The Oral Health Impact Profile (OHIP) was developed and tested in 1994, through an index of the social impact of oral alterations, and composed of forty-nine questions that describe the consequences of oral alterations ^29^. Subsequently, the need for a variety of instruments to measure OHRQoL was observed. Factor analysis and regression analysis were performed to derive a subset of the questionnaire, resulting in the OHIP-14 instrument, which maintained good reliability, validity, and accuracy ^6^. This instrument was validated in Portuguese ^23^ and includes seven dimensions ^6^. It is considered an instrument that has already been applied to root canal treatment ^7^.

Nitric oxide (NO) is a gaseous signaling molecule generated from the conversion of L-arginine to L-citrulline by nitric oxide synthases (NOS) ^30^. As it is considered an active vasodilator, NO modulates the first vascular responses of acute inflammation, when synthesized in oral diseases by activated inflammatory cells, it can regulate the functions of other cells involved in the inflammatory process ^31^. It is implicated in the pathogenesis of numerous inflammatory and autoimmune diseases ^32^. There are three isoforms of the NOS enzyme: neuronal (nNOS), inducible (iNOS), and endothelial (eNOS) ^33^, also called *NOS1*, *NOS2*, and *NOS3*, respectively. Due to the NOS's various functions, a nitrergic dysfunction seems to be a participant in several neuropsychiatric disorders ^11^ ^,^ ^12^. Inhibition of NOS can attenuate inflammatory pain ^34^ ^,^ ^35^ ^,^ ^36^, however, the molecular mechanisms underlying these effects remain to be clarified. The *NOS2* is induced by immunological and inflammatory stimuli and it is encoded by the *NOS2* gene located on chromosome 17q11.2-q12. Several studies have found an association between genetic polymorphisms in the *NOS2* gene and different phenotypes, such as lumbar disc herniation ^37^, diabetic polyneuropathies and neuropathic pain development ^13^, Achilles tendinopathy ^14^ and chronic periodontitis ^15^. Thus, the presence of an association between such variation in the *NOS2* gene with pain diseases and inflammatory diseases in other populations corroborates the results observed in the present study, which also showed an association between the rs2297518 polymorphism in *NOS2* gene and OHRQoL in five of the seven domains evaluated in OHIP-14: functional limitation, physical pain, psychological discomfort, physical disability, and psychological disability.

The group of cytokines signaling suppressors (SOCS) comprises eight proteins, called CIS and *SOCS1-SOCS7*, which are responsible for inhibiting signals in a pathway induced by cytokines that regulate several critical biological responses, including immune function and inflammatory responses ^38^. Guedes et al. ^39^ observed that the level of *SOCS1* is essential to regulate the action of some cytokines and alterations in the transcriptional activity of *SOCS1* can lead to the severity of inflammation, reporting an association between the *SOCS1*-*820* polymorphism and chronic periodontitis. Another research developed by Ghafouri-Fard et al. ^40^ revealed that together, *SOCS1*, *SOCS2*, and *SOCS3* were found to alter the circulation of patients with periodontitis, in agreement with other studies reporting abnormal expressions of genes related to the immune system in the blood of patients with periodontitis. This study observed an association between the polymorphism in the *SOCS1* gene (rs243327 and rs33977706) with the psychological domains of OHRQoL. We suggest that this fact may occur due to the pleiotropic biological capabilities of *SOCS1* and its role in regulating the suppression of cytokines that modulate the inflammatory process. Strategies to identify individuals at higher risk and inhibit the effects of these cytokines may therefore be better elucidated, as they may have a profound impact on quality of life.

Interleukins act in maintaining the functions of the innate system; therefore, the occurrence of polymorphisms in genes that encode ILs can have an extensive effect on the function of interleukins, leading to distinct pathophysiological consequences for the individual ^41^. Inflammation is mediated by gene activation, which promotes the secretion of proinflammatory cytokines and is also regulated by processes occurring in the brain ^42^. *IL-10* is a cytokine with anti-inflammatory properties that can suppress the immune response ^43^. *IL1RN*, a naturally occurring receptor antagonist, acts as an inhibitor of IL1 receptor signaling. The interleukin-1 group consists of various pro- and anti-inflammatory proteins ^44^, polymorphisms in these genes were also included in this study. The literature highlights an association between polymorphisms in genes involved in the pro- and anti-inflammatory cytokines and psychobehavioural symptoms (fatigue, depression, and cognitive impairment) in patients undergoing specific treatments ^10^ ^,^ ^25^ ^,^ ^45^. Alexander et al. ^45^ observed an association between the rs1143623 in the *IL1B* gene variation and the quality of life (QoL) in women with breast cancer. The authors reported that patients who were heterozygous or homozygous and carried at least one C allele had an increased risk for lower social well-being domain of QoL. Rausch et al. ^25^ showed that polymorphisms in several cytokine genes were associated with changes in physical functioning (i.e., *IL1B, IL10*, and *IL1RN*), mental health (*IL1RN*), emotional role functioning (*IL6*), and social functioning (*IL6, IL1RN*, and tumor necrosis factor superfamily) in a study with lung cancer survivors. Von Held et al. ^17^ reported the association between the rs17561 variation in *the IL1A* gene and OHRQoL in para-athletes with caries experience, in which the A allele in the *IL1A* gene (rs17561), in a dominant model, showed a significant association with poor psychological discomfort. Overall, our findings contradict the results of other studies that examined polymorphisms in pro- and anti-inflammatory cytokine genes and psychobehavioral symptoms to support an inflammatory basis to modulate the impact on OHRQoL. The present study showed no association between the genes *IL1A* (rs17561, rs1304037), *IL10* (rs1800871), *IL1RN* (rs9005) and OHRQoL in patients undergoing root canal treatment. Moreover, it should be noted that the differences may also be attributed to disease characteristics since the impact of the various diseases studied on quality of life is different. Thus, it is important to conduct other studies composed of different populations and diseases, aiming to better understand the association or not between these genetic factors and QoL. 

Despite this study being methodologically well-designed, some limitations can be pointed out. One is the data acquired through questionnaires that may be subject to information bias. However, measures were taken to reduce possible biases, such as the use of a validated assessment instrument, the inclusion of a sample with well-defined criteria, and adequate DNA extraction following a protocol already defined in the literature. Another limitation is the sample size. Future studies with larger samples should be conducted to confirm or refute the results found. Therefore, the present study can be considered the initial step in examining the complex relationship between genetic polymorphisms and OHRQoL in patients undergoing root canal treatment. Further studies are suggested to identify the genetic biomarkers for OHRQoL to assist health professionals in their daily activities as well as patients. In the near future, patients with a higher risk of a greater impact of root canal treatment on OHRQoL will be screened and enrolled in specific clinical follow-ups.

## Conclusion

Polymorphisms in the *NOS2* and *SOCS1* genes influenced the OHRQoL of patients undergoing root canal treatment in teeth with asymptomatic periapical periodontitis. These genes could be used as a potential biomarker for OHRQoL.
